# Benefits of Liver Volume and Serum Zinc Level Assessment for the Screening of Covert Hepatic Encephalopathy in Patients with Child–Pugh Class A Cirrhosis

**DOI:** 10.3390/diagnostics15010023

**Published:** 2024-12-25

**Authors:** Masanori Fukushima, Hisamitsu Miyaaki, Ryu Sasaki, Yasuhiko Nakao, Masafumi Haraguchi, Kosuke Takahashi, Eisuke Ozawa, Satoshi Miuma, Kazuhiko Nakao

**Affiliations:** Department of Gastroenterology and Hepatology, Nagasaki University Graduate School of Biomedical Sciences, 1-7-1 Sakamoto, Nagasaki City 852-8501, Nagasaki, Japan; miyaaki-hi@nagasaki-u.ac.jp (H.M.);

**Keywords:** covert hepatic encephalopathy, liver cirrhosis, liver volume, zinc

## Abstract

**Background/Objectives**: Covert hepatic encephalopathy (CHE) is associated with decreased quality of life. Detection of Child–Pugh class A is necessary for its early diagnosis. This study aimed to establish a simple diagnostic method of CHE in patients with Child–Pugh class A. **Methods**: One hundred patients with liver cirrhosis without overt hepatic encephalopathy and sixty-eight with liver cirrhosis and Child–Pugh class A who visited our institution were enrolled. CHE was diagnosed using number connection test B in the neuropsychiatric test (NPT). Clinical data were compared. **Results**: The liver volume/body surface area ratio (LV/BSA) was associated with CHE in patients with all-cause and Child–Pugh class A liver cirrhosis. Multiple logistic regression analysis revealed that low LV/BSA and low serum zinc (Zn) levels were significantly associated with CHE in Child–Pugh class A liver cirrhosis. The best cutoff values in the receiver operating characteristic curve analysis showed that the complication rate of CHE was 54.8% in patients with LV/BSA < 620 mL/m^2^, which was 2.9 times higher than that in patients with larger liver volume. Referring to the cutoff values for LV/BSA and Zn (<70 µg/dL), in cases with LV/BSA < 620 mL/m^2^ and Zn < 70 µg/dL, 64.2% had CHE, whereas in cases with LV/BSA ≥ 620 mL/m^2^ and Zn ≥ 70 µg/dL, 94.5% did not have CHE. **Conclusions**: Liver volume can be used as a risk assessment tool for CHE. LV/BSA and serum Zn levels are considered effective diagnostic tools for CHE, serving as alternatives to NPT in patients with Child–Pugh class A liver cirrhosis.

## 1. Introduction

Hepatic encephalopathy (HE) is a complication of liver cirrhosis that can significantly affect a patient’s quality of life (QOL), causing sleep disturbances, depression, driving difficulties, and an increased risk of falls. Covert HE (CHE), a preclinical stage of HE, is associated with these impairments. Psychometric tests are currently used to diagnose CHE; however, establishing standardized criteria is challenging, owing to variations in education, culture, cognitive function, and physical ability, particularly in older adults. Furthermore, these tests are cumbersome and time-consuming, making them difficult to administer in clinical practice. Therefore, identifying clinical markers that predict the onset of CHE is crucial.

To address these issues, the Stroop test has proven effective in reducing diagnostic time and facilitating screening [1,2]. Low albumin (Alb; ≤3.5 g/dL) and high ammonia (≥80 μg/dL) levels have been reported as clinical scores that can be easily determined from hematochemical tests alone [3].

Diagnosis of CHE by blood chemistry is simple and ideal; however, many cases identified as high-risk are Child–Pugh class B. In recent multicenter studies, the onset of CHE has been reported to correlate with a Child–Pugh score [4], and the Child–Pugh score is effective in risk discrimination. Thus, hepatic decompensation is a risk factor for CHE. Therefore, to detect CHE at an early stage, we believe it is necessary to identify cases with a high risk of developing the disease in patients with Child–Pugh class A.

Liver volume has been reported to correlate with the liver reserve capacity. Furthermore, liver volume has been reported to decrease in uncompensated cirrhosis compared with the compensated stage, and the presence or absence of overt HE correlates with liver volume [5,6]. In this study, to subdivide the liver function of patients with Child–Pugh class A, we focused on liver volume. We aimed to investigate the possibility of an association between CHE and liver volume.

## 2. Materials and Methods

### 2.1. Study Design

This study enrolled 100 consecutive patients with liver cirrhosis without history of overt HE at the Nagasaki University Hospital (Nagasaki, Japan) between 2019 and 2022. This cohort included patients with all etiologies, including alcoholic liver disease. The number connection test B (NCT-B) was used to diagnose CHE. Clinical data such as liver function, fibrosis markers, presence of esophageal varices, presence of a portosystemic shunt, and liver volume were compared. Blood tests were performed for all patients within the 7-day CHE test. Esophageal varices were defined based on a history of treatment. Computed tomography (CT) images used to measure liver volume and assess portosystemic shunts were obtained within 3 months before and after the CHE test. A portosystemic shunt was defined as having a maximum vessel diameter ≥ 5 mm. All patients with cirrhosis were treated according to the Japanese Clinical Practical Guidelines for Liver Cirrhosis.

Liver cirrhosis was identified if at least one of the following criteria was met: platelet count < 100,000/µL, presence of esophageal varices, Mac-2 binding protein glycosylation isomer (M2BPGi) > 3, fibrosis 4 (Fib4) index > 2.67, hyaluronan > 130 ng/mL, type 4 collagen 7S > 8 ng/mL, or FibroScan score > 12.5 kPa.

Next, we conducted an analysis, focusing on 68 consecutive cases of liver cirrhosis in patients with Child–Pugh class A without a history of overt HE who visited our hospital between 2019 and 2023. Given the significant effect of alcohol consumption on liver volume, patients with continuous drinking, defined as regularly drinking >20 g/day, were excluded from this study.

### 2.2. Assessment of Liver Volume

All patients underwent multiphase contrast-enhanced CT of the abdomen. Liver volume data were obtained routinely with a parallel 3D scan of the liver using SYNAPSE VINCENT^®^ image processing software ver7.0 (Fujifilm Medical Co., Tokyo, Japan). Liver volume was corrected for body surface area (LV/BSA) to account for variations in physique. The spleen volume/body surface area ratio (SPV/BSA) and liver volume/spleen volume ratio (LV/SPV) were also measured as findings suggesting portal hypertension [7].

### 2.3. CHE Diagnosis

CHE was diagnosed using computer-aided neuropsychiatric test (NPT) software ver3.1 on an iPad (Apple Inc., Cupertino, CA, USA). NPT software was developed by Otsuka Pharmaceutical Co., Ltd. (Tokyo, Japan) and provided by the Japan Society of Hepatology. NPT is composed of four subtests: NCT-A, NCT-B, digit symbol test, and block design test. In Japan, patients with liver cirrhosis are diagnosed with CHE if the results of two or more of the four subtests are abnormal [8].

Scores from the EncephalApp (Stroop-off and Stroop-on tests) and the NCT-B test can identify patients with CHE with approximately 87% accuracy and in a much shorter time than the standard psychometric HE scoring system [1]. In this study, patients who were NCT-B-positive were diagnosed with CHE.

### 2.4. Statistical Analyses

Based on CHE diagnosis, patients were divided into CHE and non-CHE groups. The significance of differences in these continuous variables across the study groups was calculated using the Mann–Whitney U test. Categorical data were analyzed using Fisher’s exact test. We calculated the odds ratio, 95% confidence interval, and *p*-values using multiple logistic regression analysis to identify factors associated with CHE. Continuous variables were dichotomized based on median values. CHE was the objective variable and clinical items were explanatory variables. The *p* values were tested against the null hypothesis of an odds ratio of 1.0 at a two-sided 5% significance level. Statistical significance was set at *p* < 0.05. Data analyses were performed using SPSS version 22.0 (IBM Corp., Armonk, NY, USA).

## 3. Results

### 3.1. CHE Correlates with Liver Functional Reserve

Of the 100 patients, 47 had CHE. When comparing the non-CHE group with the background factors, prolonged prothrombin time with international normalized ratio (PT-INR), elevated total bilirubin (TBil), and decreased Alb levels were significantly associated with CHE. These factors were determinant of the Child–Pugh score, and the Child–Pugh score was correlated with CHE. Elevated levels of fibrotic markers, including hyaluronic acid, type 4 collagen, and M2BPGi, were also noted. Additionally, a significant difference was observed in serum zinc (Zn) level, branched-chain amino acid/tyrosine molecular ratio (BTR), LV/BSA, and LV/SPV (Table 1).

**Figure 1:**
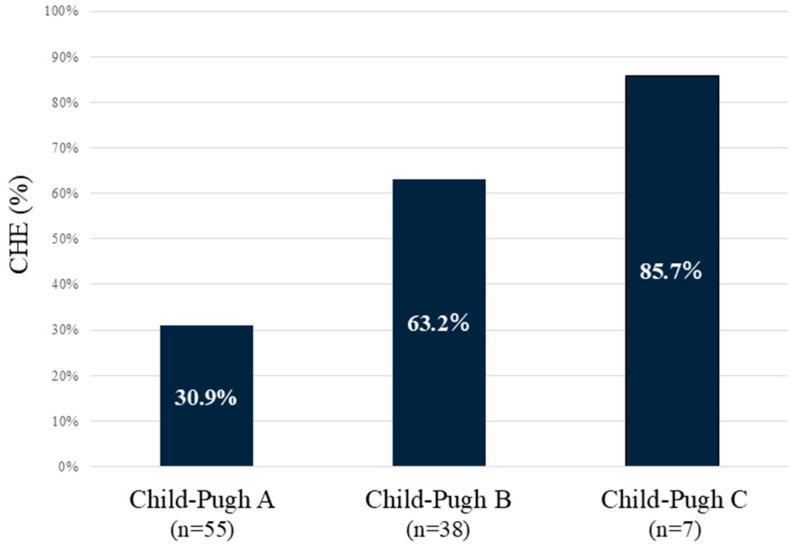
Incidence of CHE according to Child–Pugh classes. The worse the Child–Pugh class is, the higher the incidence of CHE. CHE—covert hepatic encephalopathy.

The incidence rates of CHE according to Child–Pugh classes were 30.9% in class A, 63.2% in class B, and 85.7% in class C (Figure 1). The proportion of CHE complications increased in proportion to the severity of liver function.

### 3.2. Association Between Alcohol Consumption and Liver Volume

Furthermore, to examine whether alcohol affects liver volume, we compared two groups of alcoholic liver cirrhosis during continuous drinking and during abstinence in 30 patients with alcoholic liver cirrhosis from 100 patients with liver cirrhosis. Continuous drinking was defined as regularly drinking >20 g/day. Among the groups, liver volume was larger in the continuous drinking group (606 vs. 838 mL/m^2^), with no difference in liver functional reserve among the groups (Table 2). This result indicated that individuals who consumed alcohol were to be excluded when assessing liver volume.

### 3.3. CHE in Child–Pugh Class A Correlates with Liver Volume and Serum Zn Levels

The background factors associated with CHE were compared among cases with Child–Pugh class A, excluding patients with continuous drinking (Table 3). As the liver function severity was aligned, the values for PT-INR, TBil, and Alb were comparable. We performed a multivariate analysis of the factors predicting CHE. The LV/BSA and serum Zn levels were identified as the factor contributing to the deterioration of CHE (Table 4).

**Figure 2:**
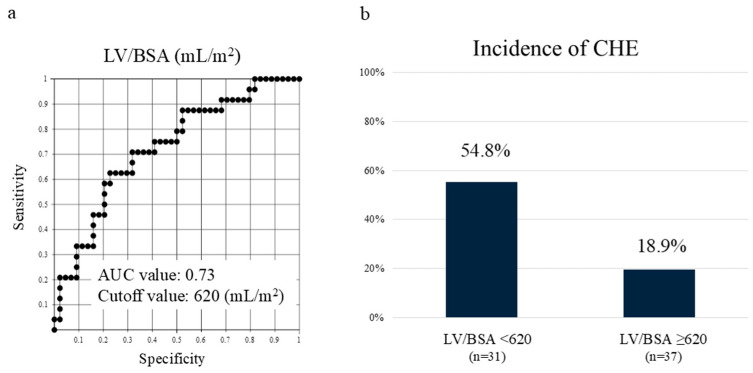
CHE in Child–Pugh class A correlates with liver volume. (**a**) Receiver operating characteristic curve of LV/BSA in Child–Pugh class A. (**b**) Setting the cutoff value at 620 mL/m^2^, the incidence of CHE was 2.9 times higher in cases below the cutoff value. LV/BSA—liver volume/body surface area; AUC—area under the curve; CHE—covert hepatic encephalopathy.

**Figure 3:**
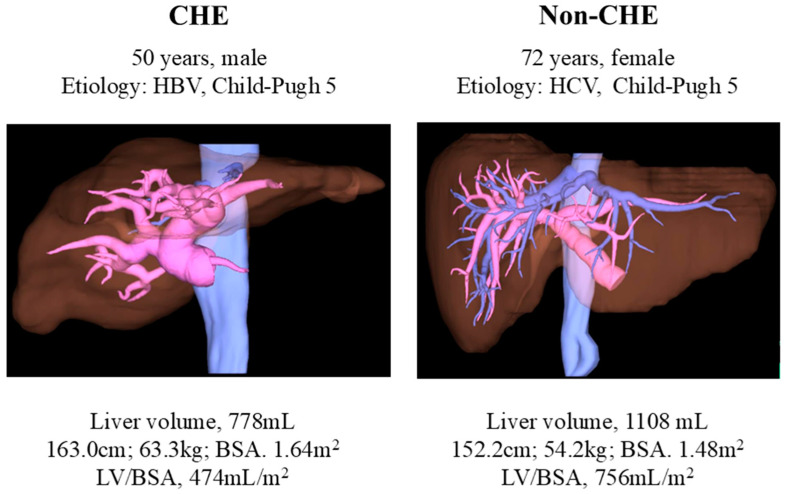
Representative image showing differences in liver volume. CHE—covert hepatic encephalopathy; HCV—hepatitis C virus; HBV—hepatitis B virus; LV/BSA—liver volume/body surface area.

Among these, the LV/BSA showed the most significant difference, with an area under the receiver operating characteristic curve of 0.74. Setting the cutoff value at 620 mL/m^2^, the incidence of CHE was 54.8%, which was 2.9 times higher in cases exceeding the cutoff value (Figure 2). The cases demonstrated significant differences in liver volume, despite similar liver functional reserves (Figure 3).

Although both groups had similar liver functional reserves, the liver volumes were significantly different. Liver volume was small in patients with CHE.

### 3.4. CHE Prediction Using Liver Volume and Serum Zn Levels

**Figure 4:**
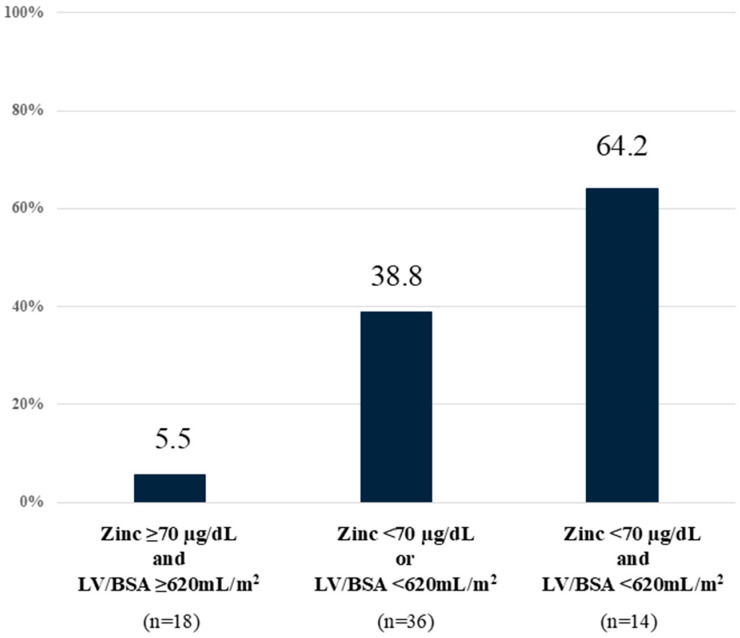
CHE prediction using liver volume and serum zinc levels. Low LV/BSA (<620 mL/m^2^) and low zinc level (<70 µg/dL) were associated with high incidence of CHE. CHE—covert hepatic encephalopathy; LV/BSA—liver volume/body surface area.

The cutoff value for serum Zn levels was set at 70 µg/dL. The CHE prevalence was 64.2% (9/14) in patients with low LV/BSA (<620 mL/m^2^) and low Zn level (<70 µg/dL), 38.8% (14/36) in patients with low LV/BSA or low Zn level, and 5.5% (1/18) in patients with high LV/BSA and high Zn level (Figure 4). This suggests that, in cases of LV/BSA ≥ 620 mL/m^2^ and Zn ≥ 70 µg/dL, the possibility of CHE is extremely low.

## 4. Discussion

This study is one of the first attempts to assess the liver volume to stratify the risk of CHE development in patients with liver cirrhosis. We found that in patients with Child–Pugh class A and no difference in liver functional reserve, CHE was more commonly observed in those with smaller liver volumes. Liver volume, measurable readily through imaging modalities such as CT, provides an objective measure unaffected by patient background compared with NPT. In addition, the addition of serum Zn levels may enable the diagnosis of CHE with higher accuracy. We believe that this method has clinical significance and can serve as a valuable tool for risk assessment in patients with liver cirrhosis.

The prevalence of CHE in patients with cirrhosis has been reported to be 30–50% [9,10]. The gold standard in the diagnosis of CHE is the psychometric hepatic encephalopathy score test, which includes five tests: digit symbol test; NCT A (letters) and B (letters and numbers); serial dotting test; line tracing test [11]. However, the line tracing and serial dotting tests are not common in Japan, and applying them is difficult. Therefore, in Japan, the NPT was developed to perform eight tests: figure design test; digit symbol test; block design test; reaction time A, B, and C tests [12]. Performing all these tests originally required 30–40 min. However, Kawaguchi et al. reported that reducing the number of NPTs from eight to four—the NCT-A, NCT-B, digit symbol test, and block design test—did not affect the diagnosis of CHE, enabling CHE diagnosis within 15–20 min. Therefore, this approach is currently used to diagnose CHE [8].

The liver volume is usually smaller in patients with cirrhosis than in healthy individuals. Liver volume assessment is utilized in preoperative planning for liver resection or transplantation, and the postoperative remnant liver volume is correlated with postoperative outcomes [13]. Liver volume has been identified as an independent predictor of prognosis in patients with cirrhosis, separate from the model for end-stage liver disease (MELD) score [14]. Additionally, liver volume also correlates with the Child–Pugh score [15,16], suggesting its potential as an independent indicator of liver functional reserve, akin to MELD and Child–Pugh scores. Furthermore, recent multicenter studies have reported an association between CHE onset and Child–Pugh score [4]. Although the Child–Pugh score serves as a useful score for assessing CHE risk, a one-point difference in the Child–Pugh score indicates a significant difference in liver function. Therefore, we focused on the liver volume as an item that can express the difference between Child–Pugh scores in more detail.

Liver volume was correlated with body surface area, and a method for calculating the standard liver volume using BSA was applied [17,18]. In the Urata formula, the standard liver volume is expressed as 706.2 × BSA + 2.4 [18]. Therefore, in this study, the LV/BSA ratio was used to correct for differences in liver volume due to differences in body size. Liver volume increases during alcohol consumption [19]. In our study, compared with the abstinence group, liver volume was larger in the continuous drinking group with no difference in liver functional reserve; therefore, when liver volume is used as an indicator of liver functional reserve, ensuring that the patient is not drinking alcohol is crucial.

Ammonia metabolism, which contributes to HE, requires the liver’s ability to process ammonia. If the liver cannot process it, glutamate is used to process ammonia as a compensatory measure, resulting in increased protein catabolism in skeletal muscle and a deficiency associated with branched-chain amino acid consumption, which causes a decrease in the BTR [20]. The mechanism of ammonia metabolism is initially a decrease in BTR, followed by a decrease in Alb, and finally an increase in ammonia. Patients with Child–Pugh grade A had no decrease in the BTR or Alb levels, making risk assessment with ammonia difficult. Therefore, LV/BSA, which was found to be a predictor of CHE development in this study, can predict CHE risk earlier than Alb and ammonia, which have been previously reported.

In this study, low liver volume and serum Zn level were risk factors of CHE in patients with Child–Pugh grade A. Zn deficiency frequently occurs in patients with liver cirrhosis [21]. Zn deficiency is attributed to impaired absorption in the gastrointestinal tract associated with cirrhosis and increased urinary excretion due to the enhanced relative binding of amino acids and Zn as a consequence of low albumin levels. Zn is essential for the urea cycle that metabolizes ammonia, and elevated ammonia resulting from Zn deficiency is postulated to contribute to HE [22,23]. Consequently, Zn levels decrease prior to the elevation of ammonia levels, rendering Zn a potential biomarker for the early detection of CHE. In a cohort with 73.8% Child–Pugh class A, Soma et al. reported that Zn < 60 μg/dL was an independent risk factor for CHE. This finding corroborates the observation that in the Child–Pugh class A-only cohort of this study, Zn levels < 70 μg/dL constituted a risk factor [24].

In living donor liver transplantation, at least 35% of the donor’s remaining liver is required [25], and at least 40% of the standard liver volume is required for the recipient’s graft [18,26]. If 40% of the standard liver volume is required, the LV/BSA would be approximately 300 mL/m^2^. The cutoff value for the risk of CHE in our study was 620 mL/m^2^, which is a considerably small liver volume. However, we believe that liver transplant grafts and donor liver remnants function without complications because the liver is composed of healthy hepatocytes. Therefore, we believe that the function of a cirrhotic liver may be reduced to half of that of a normal liver. Serum Zn level and liver volume may be useful in determining liver function in patients with cirrhosis before abnormalities in Alb, PT, and TBil are observed, as expressed by the Child–Pugh classification. In this study, hyaluronic acid levels also showed a significant difference in univariate analysis, suggesting an association with CHE among fibrosis markers. The fibrosis marker hyaluronic acid differs from other fibrosis markers in that it is produced by astrocytes and is characterized by degradation by sinusoidal endothelial cells [27,28]. High levels of hyaluronic acid in the CHE group may reflect functional defects in terms of reduced liver degradation.

In recent years, there have been reports on the utility of novel serum biomarkers such as interleukin 6 and glial fibrillary acidic protein that have been utilized for the diagnosis of CHE [29,30]. The strength of liver volume evaluation in the diagnosis of CHE lies in its utilization of CT, eliminating the need for additional tests and enabling assessment independent of factors such as the patient’s mental state or educational background.

One limitation of this study is that it was a single-center retrospective analysis with a small number of cases. Due to the small number of cases, evaluation by etiology was not possible in this study. Differences may exist in the appropriate cutoff value for each etiology in liver volume; therefore, further accumulation of cases and additional analysis are required in the future. Additionally, verification of long-term outcomes, such as progression to overt HE, in patients diagnosed with CHE is required.

## 5. Conclusions

Despite the limitation of this study, this is the first to describe the relationship between liver volume and CHE. In this study, liver volume was used as an index reflecting the liver functional reserve capacity and as a risk assessment tool for the development of CHE. Our study indicates that identifying cases with small liver volumes is beneficial in efficiently detecting the risk of CHE in patients with Child–Pugh class A liver cirrhosis. Furthermore, the integration of serum Zn level measurements enhances the diagnostic accuracy for CHE. LV/BSA and serum Zn levels can be considered as effective diagnostic tools for CHE and can act as alternatives to the current diagnostic tools.

## Figures and Tables

**Table 1:** Characteristics of the CHE and non-CHE groups in liver cirrhosis.

	Total (*n* = 100)	CHE (*n* = 47)	Non-CHE (*n* = 53)	*p* Value
Age (years)	68 [61–73]	65 [58–73]	69 [63–73]	ns
Sex				ns
Male	62	28	34	
Female	38	19	19	
BMI (kg/m^2^)	23.1 [20.7–25.7]	23.8 [20.8–27.2]	22.9 [20.6–25.2]	ns
Etiology				ns
HCV	19	7 (15)	12 (23)	
HBV	16	8 (17)	8 (15)	
MASH	24	10 (21)	14 (26)	
AL	30	14 (30)	16 (30)	
Other	11	8 (17)	3 (6)	
Child–Pugh grade				0.0001
A	55	17	38	
B	38	24	14	
C	7	6	1	
Plt (10^4^/µL)	9.4 [6.1–13.0]	8.5 [5.4–12.0]	10.2 [7.5–13.4]	ns
PT-INR	1.18 [1.08–1.30]	1.24 [1.10–1.42]	1.13 [1.06–1.23]	0.002
TBil (mg/dL)	1.1 [0.8–1.6]	1.3 [0.9–2.0]	1.0 [0.7–1.4]	0.004
Alb (g/dL)	3.3 [2.7–3.7]	3.0 [2.4–3.5]	3.5 [2.9–3.8]	0.002
Na (mmol/L)	140 [138–141]	139 [138–141]	140 [138–141]	ns
eGFR (mL/min/1.73 m^2^)	69.0 (55.3–77.9)	70.6 (49.2–79.6)	66.3 (57.1–76.2)	ns
Hyaluronic acid (ng/mL)	254 [147–474]	338 [209–715]	191 [116–314]	0.0003
Type 4 collagen 7S (ng/mL)	8.7 [6.3–12.0]	10.2 [7.3–14.1]	7.4 [5.9–10.4]	0.003
M2BPGi	3.83 [2.10–7.31]	5.95 [3.13–14.13]	3.18 [1.68–4.75]	0.0002
NH_3_ (µg/dL)	67 [45–97]	69 [44–128]	66 [46–95]	ns
Zinc (µg/dL)	58 [44–71]	53 [39–66]	65 [53–72]	0.002
Fib4	5.6 [3.7–7.3]	5.9 [4.1–7.7]	5.1 [3.6–8.2]	ns
BTR	4.1 [3.1–5.5]	3.6 [2.8–4.9]	4.7 [3.7–5.6]	0.007
LV/BSA (mL/m^2^)	591 [476–759]	530 [424–700]	691 [520–779]	0.002
SPV/BSA (mL/m^2^)	207 [126–327]	256 [136–381]	182 [125–282]	ns
LV/SPV	3.1 [1.7–5.6]	2.1 [1.6–4.1]	3.6 [2.3–5.8]	0.012
Esophageal varices	54	26 (55)	28 (53)	ns
Portosystemic shunt	28	14 (30)	14 (26)	ns
Sarcopenia	22	10 (21)	12 (23)	ns
Drug therapy				ns
BCAA	55	18	27	
Lactulose	18	5	13	
Rifaximin	12	3	9	

Data are shown as medians [interquartile ranges], numbers, or numbers (percentages). CHE—covert hepatic encephalopathy; BMI—body mass index; HCV—hepatitis C virus; HBV—hepatitis B virus; MASH—metabolic dysfunction-associated steatohepatitis; AL—alcoholic liver; Plt—platelets; PT-INR—prolonged prothrombin time with international normalized ratio; TBil—total bilirubin; Alb—albumin; Na—sodium; eGFR—estimated glomerular filtration rate; M2BPGi—Mac-2 binding protein glycosylation isomer; NH_3_—ammonia; Fib4—fibrosis 4; BTR—branched-chain amino acid/tyrosine molecular ratio; LV/BSA—liver volume/body surface area; SPV/BSA—spleen volume/body surface area; LV/SPV—liver volume/spleen volume; BCAA—branched-chain amino acid; ns—not significant.

**Table 2:** Characteristics of the abstinence and continued drinking groups in alcoholic liver cirrhosis.

	Abstinence (*n* = 16)	Continued Drinking (*n* = 14)	*p* Value
Age (years)	64.5 [61.0–70.5]	60.5 [54.0–64.0]	ns
Sex			ns
Male	16	11	
Female	0	3	
BMI (kg/m^2^)	23.3 [21.8–25.7]	21.9 [20.0–25.8]	ns
Child–Pugh score	6.5 [5.0–8.5]	6.5 [6.0–9.0]	ns
Plt (10^4^/µL)	7.2 [4.8–13.1]	9.2 [6.6–12.1]	ns
PT-INR	1.13 [1.09–1.39]	1.19 [1.06–1.32]	ns
AST (U/L)	25 [20–34]	50 [39–68]	0.002
ALT (U/L)	18 [16–26]	25 [21–34]	0.04
γ-GTP (U/L)	30 [21–82]	203 [52–313]	0.006
TBil (mg/dL)	1.1 [0.9–1.7]	1.5 [0.9–3.2]	ns
Alb (g/dL)	3.2 [2.7–3.8]	3.0 [2.8–3.4]	ns
eGFR (mL/min/1.73 m^2^)	64.4 (54.5–76.2)	82.7 (75.7–86.8)	0.007
Hyaluronic acid (ng/mL)	218 [136–524]	325 [91–950]	ns
Type 4 collagen 7S (ng/mL)	10.4 [7.6–12.0]	11.3 [7.7–14.7]	ns
M2BPGi	4.3 [3.0–6.7]	5.6 [3.4–10.5]	ns
NH_3_ (µg/dL)	100 [60–127]	69 [63–86]	ns
Zinc (µg/dL)	59 [42–70]	49 [43–56]	ns
Fib4	5.1 [3.0–8.3]	6.8 [4.3–9.3]	ns
BTR	4.3 [3.4–5.7]	3.2 [2.9–4.9]	ns
LV/BSA (mL/m^2^)	559 [477–701]	832 [671–1043]	0.004
SPV/BSA (mL/m^2^)	289 [143–395]	198 [147–249]	ns
LV/SPV	2.1 [1.3–4.6]	3.4 [2.4–6.0]	0.04
Esophageal varices	12 (75)	10 (71)	ns
Portosystemic shunt	6 (38)	6 (43)	ns
Sarcopenia	4 (25)	1 (8)	ns
Drug therapy			ns
BCAA	10	6	
Lactulose	5	0	
Rifaximin	3	0	

Data are shown as medians [interquartile ranges], numbers, or numbers (percentages). BMI—body mass index; Plt—platelets; PT-INR—prolonged prothrombin time with international normalized ratio; AST—aspartate aminotransferase; ALT—alanine transaminase; γ-GTP—γ-glutamyl transpeptidase; TBil—total bilirubin; Alb—albumin; eGFR—estimated glomerular filtration rate; M2BPGi—Mac-2 binding protein glycosylation isomer; NH_3_—ammonia; Fib4—fibrosis 4; BTR—branched-chain amino acid/tyrosine molecular ratio; LV/BSA—liver-body surface area ratio; SPV/BSA—spleen volume/body surface area ratio; LV/SPV—liver/spleen volume ratio; BCAA—branched-chain amino acid; ns—not significant.

**Table 3:** Characteristics of the CHE and non-CHE groups in Child–Pugh class A liver cirrhosis.

	Total (*n* = 68)	CHE (*n* = 24)	Non-CHE (*n* = 44)
Age (years)	71.5 (67.0–77.5)	71.0 (67.0–77.5)	71.5 (67.0–77.5)
Sex			
Male	41	16	25
Female	27	8	19
BMI (kg/m^2^)	22.8 (20.7–25.0)	22.3 (20.5–24.6)	22.9 (21.2–25.0)
Etiology			
HCV	24 (35)	8 (33)	16 (36)
HBV	15 (22)	8 (33)	7 (16)
MASH	18 (26)	5 (21)	13 (30)
AL	6 (9)	1 (4)	5 (11)
Other	5 (7)	2 (8)	3 (7)
Child–Pugh score			
5	44	15	29
6	24	9	15
Plt (10^4^/µL)	11.6 [8.8–15.4]	9.6 [8.0–13.0]	12.9 [9.7–17.3]
PT-INR	1.09 [1.03–1.17]	1.09 [1.03–1.20]	1.09 [1.02–1.17]
TBil (mg/dL)	1.0 [0.7–1.1]	1.0 [0.7–1.4]	0.9 [0.7–1.1]
Alb (g/dL)	3.8 [3.5–4.0]	3.7 [3.2–4.0]	3.8 [3.5–4.0]
eGFR (mL/min/1.73 m^2^)	69.7 (56.6–77.3)	72.2 (58.7–78.2)	65.3 (56.6–73.6)
Hyaluronic acid (ng/mL)	191 [110–275]	213 [177–340]	172 [100–252]
Type 4 collagen 7S (ng/mL)	6.3 [4.5–7.8]	6.6 [4.9–8.7]	6.2 [4.4–7.5]
M2BPGi	2.0 [1.1–3.3]	2.2 [1.2–3.3]	1.7 [1.1–3.4]
NH_3_ (µg/dL)	48 [39–70]	43 [33–59]	48 [40–73]
Zinc (µg/dL)	70 [62–82]	65 [60–76]	73 [64–83]
Fib4	4.1 [3.0–6.2]	4.7 [3.3–6.3]	4.0 [2.7–6.1]
BTR	5.5 [4.4–6.4]	5.3 [3.9–6.9]	5.5 [4.4–6.4]
LV/BSA (mL/m^2^)	668 [513–758]	550 [488–706]	726 [574–797]
SPV/BSA (mL/m^2^)	155 [85–279]	195 [87–287]	139 [83–266]
LV/SPV	4.8 [2.8–7.4]	3.7 [1.9–6.9]	5.1 [3.1–7.8]
Esophageal varices	25 (37)	9 (38)	16 (36)
Portosystemic shunt	10 (15)	3 (13)	7 (16)
Sarcopenia	17 (25)	5 (22)	12 (27)
Drug therapy			
BCAA	15	8	7
Lactulose	6	4	2
Rifaximin	1	1	0

Data are shown as medians [interquartile ranges], numbers, or numbers (percentages). CHE—covert hepatic encephalopathy; BMI—body mass index; HCV—hepatitis C virus; HBV—hepatitis B virus; MASH—metabolic dysfunction-associated steatohepatitis; AL—alcoholic liver; Plt— platelets; PT-INR—prolonged prothrombin time with international normalized ratio; TBil—total bilirubin; Alb—albumin; Na—sodium; eGFR—estimated glomerular filtration rate; M2BPGi—Mac-2 binding protein glycosylation isomer; NH_3_—ammonia; Fib4—fibrosis 4; BTR—branched-chain amino acid/tyrosine molecular ratio; LV/BSA—liver volume/body surface area ratio; SPV/BSA—spleen volume/body surface area ratio; LV/SPV—liver-spleen volume ratio; BCAA—branched-chain amino acid.

**Table 4:** Factors contributing to CHE in Child–Pugh class A liver cirrhosis (*n* = 68).

		Univariate Analysis		Multivariate Analysis	
		OR (95% CI)	*p* Value	OR (95% CI)	*p* Value
Age (years)	≥71	0.76 (0.280–2.062)	0.589		
Sex	Female	0.658 (0.233–1.856)	0.429		
BMI (kg/m^2^)	≥23	0.782 (0.287–2.136)	0.631		
Etiology	HCV or HBV	2.000 (0.711–5.626)	0.188		
Child–Pugh score	6	1.160 (0.412–3.266)	0.778		
Plt (10^4^/µL)	<12	1.825 (0.661–5.044)	0.245		
PT-INR	≥1.1	0.782 (0.287–2.136)	0.631		
TBil (mg/dL)	≥1.0	1.294 (0.477–3.509)	0.621		
Alb (g/dL)	<3.8	1.388 (0.474–4.059)	0.549		
eGFR (mL/min/1.73 m^2^)	≥70	2.685 (0.973–7.404)	0.056		
Hyaluronic acid (ng/mL)	≥190	2.976 (1.008–8.786)	0.048	3.291 (0.947–11.440)	0.06
Type 4 collagen 7S (ng/mL)	≥6.3	1.257 (0.449–3.523)	0.663		
M2BPGi	≥2.0	1.825 (0.644–5.186)	0.257		
NH_3_ (µg/dL)	≥50	0.806 (0.291–2.231)	0.677		
Zinc (µg/dL)	<70	2.976 (1.008–8.786)	0.048	4.578 (1.225–11.440)	0.023
Fib4	≥4.0	1.485 (0.531–4.155)	0.451		
BTR	≤5.5	0.873 (0.312–2.446)	0.796		
LV/BSA (mL/m^2^)	<670	3.508 (1.208–10.184)	0.021	7.715 (1.965–30.293)	0.003
SPV/BSA (mL/m^2^)	≥155	1.321 (0.477–3.659)	0.592		
LV/SPV	≥4.8	0.531 (0.189–1.494)	0.23		
Esophageal varices	+	1.085 (0.383–3.072)	0.878		
Portosystemic shunt	+	0.755 (0.176–3.234)	0.705		
Sarcopenia	+	0.741 (0.225–2.441)	0.621		
Drug therapy	+	2.250 (0.717–7.059)	0.164		

CHE—covert hepatic encephalopathy; OR—odds ratio; CI—confidence interval; BMI—body mass index; Plt—platelets; PT-INR—prolonged prothrombin time with international normalized ratio; TBil—total bilirubin; Alb—albumin; Na—sodium; eGFR—estimated glomerular filtration rate; M2BPGi—Mac-2 binding protein glycosylation isomer; NH_3_—ammonia; Fib4—fibrosis 4; BTR—branched-chain amino acid/tyrosine molecular ratio; LV/BSA—liver volume/body surface area ratio; SPV/BSA—spleen volume/body surface area ratio; LV/SPV—liver volume/spleen volume ratio.

## Data Availability

The data presented in this study are available on request from the corresponding author. The data are not publicly available, as they contain sensitive medical information.

## Abbreviations

The following abbreviations are used in this manuscript:

Alb	Albumin
AL	Alcoholic liver
ALT	Alanine transaminase
AST	Aspartate aminotransferase
BCAA	Branched-chain amino acid
BMI	Body mass index
BTR	Branched-chain amino acid-to-tyrosine molecular ratio
CHE	Covert hepatic encephalopathy
CI	Confidence interval
CT	Computed tomography
eGFR	Estimated glomerular filtration rate
Fib4	Fibrosis-4 index
γ-GTP	γ-Glutamyl transpeptidase
HBV	Hepatitis B virus
HCV	Hepatitis C virus
HE	Hepatic encephalopathy
LV/BSA	Liver volume-to-body surface area ratio
LV/SPV	Liver volume-to-spleen volume ratio
M2BPGi	Mac-2 binding protein glycosylation isomer
MASH	Metabolic dysfunction-associated steatohepatitis
MELD	Model for end-stage liver disease score
Na	Sodium
NCT-B	Number connection test B
NH_3_	Ammonia
NPT	Neuropsychiatric test
OR	Odds ratio
Plt	Platelets
PT-INR	Prothrombin time/international normalized ratio
QOL	Quality of life
SPV/BSA	Spleen volume-to-body surface area ratio
TBil	Total bilirubin
TLA	Three-letter acronym
Zn	Zinc
